# Health expenditures of asthma-COPD overlap in Northern Jordan

**DOI:** 10.1371/journal.pone.0257566

**Published:** 2021-09-21

**Authors:** Shoroq M. Altawalbeh, Bushra Hijazi, Lara Kufoof, Iman A. Basheti

**Affiliations:** 1 Department of Clinical Pharmacy, School of Pharmacy, Jordan University of Science and Technology, Irbid, Jordan; 2 Plan International Organization, Amman, Jordan; 3 Department of Clinical Pharmacy & Therapeutics, Faculty of Pharmacy, Applied Science Private University, Amman, Jordan; Clinic for Infectious and tropical diseases, Clinical centre of Serbia, SERBIA

## Abstract

**Objective:**

To investigate the characteristics and medical expenditures of patients with Asthma- chronic obstructive pulmonary disease (COPD) overlap (ACO) compared to asthma and COPD patients.

**Methods:**

This was a retrospective cohort study involving patients diagnosed with ACO, asthma or COPD as of January 2016. Medical records for patients attending King Abdullah University Hospital (KAUH), in northern Jordan, during the years 2015–2016 were used to identify eligible patients and all relevant clinical characteristics. Both respiratory and all-cause charges were extracted from KAUH billing system during the year 2016. Total, inpatient, outpatient, and pharmacy charges were described and compared across the three disease categories. Charges were measured in Jordanian Dinar (JOD, equal to 1.41 US Dollar).

**Results:**

Of a total of 761, 87 ACO patients, 494 asthmatic patients and 180 COPD patients were identified and included in this study. The average total respiratory-related charges were significantly higher in patients with ACO compared to patients with asthma (601.4 versus 354.3 JODs; P value < 0.001). Average all-cause charges were higher in case of ACO and COPD compared to patients with asthma (1830.8 and 1705.4 versus 1251.7 JODs; P value < 0.001). ACO was a significant predictor of higher respiratory and all-cause related charges. Respiratory charges were also higher in older patients and those with higher disease severity.

**Conclusions:**

ACO is a risk factor for incurring higher health expenditures in Jordan. Higher respiratory expenditures are also associated with older ages and higher disease severity.

## 1 Introduction

Asthma and chronic obstructive pulmonary disease (COPD) are common respiratory diseases that have distinctive clinical and pathophysiological features [1]. Asthma-COPD overlap (ACO) is assigned to patients presenting with overlapping symptoms of asthma and COPD [2]. Having airway hyper-responsiveness, coexisting with incompletely reversible airway obstruction, indicates the presence of ACO [3, 4]. The global prevalence of ACO has been estimated to be around 2.0% of the general population and varied across population-based studies from 3.2 to 51.4% among patients with asthma, and from 13.0 to 55.7% among patients with COPD [5]. These variations in prevalence across the countries are due to variations in COPD and asthma diagnostic criteria, in addition to the population demographics including age, gender, and smoking status [6].

Asthma and COPD are major airway diseases that independently contribute to high socioeconomic burden [7, 8]. As for ACO, it has been associated with high risk of exacerbations and poor health outcomes, and hence became of concern all over the world [9, 10]. Moreover, patients with ACO have been shown to have greater health care costs compared with asthma or COPD patients alone [11–13]. In fact, subjects with ACO have been systematically excluded from major clinical trials, and accordingly, limited evidence about their treatment has been published [14, 15]. To date, diagnosing and managing ACO is still challenging and need further efforts and evidence-based management guidelines [16, 17].

In the Middle East, patients with ACO are still poorly characterized and their resource utilization has not been well appreciated compared to patients with asthma or COPD only. ACO patients in Jordan are usually treated in hospitals either as inpatients, in the emergency department (ED) or in outpatient clinics in stable conditions. Hospital expenditures are expected to capture the major components of ACO patient expenditures in Jordan. This study was designed to enhance the current state of knowledge of the burden associated with ACO, asthma, and COPD in Jordan by unveiling the characteristics of ACO patients, their healthcare utilization, and medical costs and comparing them to asthma and COPD patients.

## 2 Methods

### 2.1 Study design and sample

This retrospective cohort study was conducted during the period from January 1, 2016 to December 31, 2016. Data from the year before (2015) were used to confirm the diagnosis and to extract relevant baseline data. Patient’s computerized medical records at King Abdullah University Hospital (KAUH) were searched by a specialized information technology personnel to identify all patients who were treated with any respiratory related medications including inhaled corticosteroids (ICSs), long-acting beta-adrenoreceptor agonists (LABAs), and anticholinergic agents (ATC), or combination(s) during 2015, and aged 40 years and older as of January 2016.

The medical records for all identified patients were then reviewed manually by the research team to determine respiratory diagnoses that were classified as asthma, COPD or ACO as of January 2016. This classification was primarily based on physician diagnosis as documented in physician notes. Patients were excluded from the study if they did not have data available for the whole 2016 year such as those died during 2016, if they had other respiratory problems such as lung cancer, lung fibrosis, sarcoidosis, bronchiectasis and cystic fibrosis, or if they had any of the following disorders: psychiatric disorders, end stage organ damage (such as end stage renal dialysis and liver cirrhosis), autoimmune diseases (such as systemic lupus erythematosus and rheumatoid arthritis), anatomical heart disorders, and Alzheimer. This research was approved by the Institutional Review Board (IRB) Committee at KAUH (Ref number: 32/98/2016).

### 2.2 Data source

Medical records and pharmacy data for patients attending the KAUH, located in northern Jordan, were used to identify eligibe patients to be included in the study, in addition to all relavent study data (as explained below). Medical records (electronic and paper files) were used to confirm respiratory diagnoses of patients, and to extract information about their demographics, comorbidities, plumonary function tests, and reproted exacerbations. Pharmacy data were used to identify respiratory related treatments used, and other medications prescribed for study patients.

The billing system at the KAUH was used to extract all incurred expenditures for the study sample during 2016, including charges of admissions, outpatient visits and ED visits in total and by service components.

The medication components of these expenditures were extracted in details with the amounts and associated gross charges.

### 2.3 Study outcomes and patients’ variables

#### 2.3.1 Study outcomes

Patient expenditures were classified as respiratory and non-respiratory related charges. Inpatient respiratory hospitalizations were identified using the International Classification of Diseases, 10^th^ version (ICD-10) codes related to diseases of the respiratory system (J00-J99). Respiratory related outpatient visits were classified based on the specialty of annotated physicians. The ED visits were determined if the patients were prescribed bronchodilators or systematic corticosteroids that were related to the respiratory exacerbations during their ED visits.

Charges were described for the inpatient and outpatient settings in total, and by the service provided (charge components). For each inpatient admission, charge per day was calculated by deviding the total charge by the length of hospital stay in days. Inpatient charge components were classified as drugs, labs, radiology, residency, supervision, procedures, consumables, operation fees, anesthesia, room and equipment fees and other miscellaneous fees. The outpatient charge components included drugs, labs, radiology, clinic visit, procedures, and consumables.

Medication charges were retrieved in detail (with drug names, quantities, gross charges, and dates of prescription). Drugs were further classified as respiratory and non-respiratory medications. Respiratory related charges and all-cause charges were described and compared between asthma, COPD and ACO patients. Charges were measured in Jordanian Dinar (JOD, equal to 1.41 US Dollar).

#### 2.3.2 Patient variables

Covariates were obtained and extracted from the patients’ medical records, which included patients’ characteristics, in addition to characteristics of their respiratory diseases. Patients’ characteristics included age, gender, weight, and height (to calculate body mass index (BMI)). Characteristics of the respiratory diseases included the respiratory disease duration, co-morbidities, and smoking status (smoker/ex-smoker, or nonsmoker).

Severity of lung function impairment was categorized based on the most recent forced expiratory volume in one second (FEV^1^)% as normal (FEV^1^% ≥80), mild to moderately severe (FEV^1^% = 50–79) and severe to very severe (FEV^1^% < 50) [18]. Respiratory disease severity/control was determined based on both pulmonary function impairment and the presence of respiratory exacerbations in 2015. Severe disease was considered if FEV^1^% was <50 or if the patient had one or more respiratory exacerbations in 2015; mild-moderate disease was considered if FEV^1^% ≥ 50, or if the patient had no respiratory exacerbations in 2015 [19].

### 2.4 Statistical analysis

Data entry and analyses were conducted using STATA version 14 (StataCorp. 2015. Stata. Statistical Software: Release 14. College Station, TX: StataCorp LP.).

Descriptive statistics, such as percentages and arithmetic means with standard deviations, were calculated to describe patient characteristics for the total sample and by respiratory disease. Patient characteristics were compared between asthma, COPD and ACO patients using unadjusted bivariable tests (chi-square for categorical variables, t-test for continuous variables).

Respiratory and all-cause charges incurred were described with means and standard deviations per patient and per visit (inpatient and outpatient) and were compared between the three disease categories using Kruskal-Wallis tests. Log transformation of charges was used to evaluate predictors of all-cause and respiratory related charges. Variables included in these regression models were selected using backward stepwise process with P<0.2 to stay. Average charges of respiratory related admissions and outpatient visits were aggregated and described by services provided (charge components). Respiratory-related medication charges were analyzed and described on the patient level. Respiratory medication charges and all-cause medication charges were compared between the three disease categories.

## 3 Results

### 3.1 Patient characteristics

In total, 494 asthmatic patients, 180 COPD patients and 87 ACO patients were identified during the study time and were included in the study. Table 1 describes the demographic characteristics of the study sample for asthma, COPD and ACO patients; the average age was close for COPD and ACO patients (65.1±10.1 and 64.3±11.7 years) than that for asthma patients (58.1±11.9 p value = <0.001). The majority of COPD (88.9%) and ACO (69%) patients were males compared to only 27.7% of asthma patients being males. Similarly, smoking was more prevalent among the COPD and ACO patients compared to asthmatic patients. Cardiovascular (CV) comorbid conditions were prevalent among the study sample regardless of the nature of condition (asthma = 64.4%, COPD = 76.7%, ACO = 72.4%).

**Table 1:** Study population characteristics in adults with asthma, COPD and ACO.

Variable	Asthma	COPD	ACO	P value^a^
	n = 494 (64.9%)	n = 180 (32.6%)	n = 87 (11.4%)	
**Age, years (SD)**	58.1(11.9)	65.0 (10.1)	64.3(11.7)	<0.001
**Age ≥ 65**	146 (29.6)	96 (53.3)	44 (50.6)	<0.001
**Male gender**	137 (27.7)	160 (88.9)	60 (69.0)	<0.001
**BMI**				
< 25	34 (16.67)	31(29.8)	7(14.9)	0.012
25–29.9	68 (33.3)	40 (38.5)	16(34.04)	
≥ 30	102 (50.0)	33(31.73)	24(51.06)	
**Smoking status**				
Non-smoker	241(71.7)	7 (4.1)	12 (14.6)	<0.001
Ex-smoker	40 (11.9)	59 (34.9)	27 (32.9)	
Smoker	55 (17.4)	103 (61)	43 (52.4)	
**GERD**	50 (10.1)	11 (6.1)	6 (6.9)	0.214
**HF**	33 (6.7)	27 (15)	18 (20.7)	<0.001
**IHD**	114 (23.1))	87 (48.3)	40 (46.0)	<0.001
**HTN**	282 (57.2)	120 (66.7)	56 (64.4)	0.060
**AF**	14 (2.8)	10 (5.6)	3 (3.5)	0.240
**Stroke**	19 (3.9)	22 (12.2)	4 (4.6)	<0.001
**DM**	186 (37.7)	75 (41.7)	36 (41.4)	0.583
**CV comorbidities** ^b^	318 (64.4)	138 (76.7)	63 (72.4)	0.007
FEV^1^**%**				
≥ 80	46 (41.8)	11 (16.2)	8 (21.6)	0.005
50–79	46 (41.8)	39 (57.4)	19 (51.4)	
<50	18 (16.4)	18 (26.5)	10 (27.0)	
**Maintenance treatments used**				
ATC	5 (1.01)	35(19.4)	2 (2.3)	<0.001
ICS+LABA	442 (89.5)	63 (35.0)	40 (46.0)	
ICS+LABA +ATC	47 (9.5)	82 (45.6)	45 (51.7)	
**Severe pulmonary disease**	48 (9.7)	36 (20.0)	22 (25.3)	<0.001
**Respiratory exacerbations in 2015**	36 (7.3)	26 (14.4)	15 (17.2)	0.002

Abbreviations: BMI: body mass index, COPD: chronic obstructive pulmonary disease, GERD: gastroesophageal reflux disease, HF: heart failure, IHD: ischemic heart disease, HTN: hypertension, AF: atrial fibrillation, DM: diabetes mellitus, CV: cardiovascular, FEV^1^: forced expiratory volume in one second, ICS: inhaled corticosteroid, LABA: long-acting beta-adrenoreceptor agonist, ATC: anticholinergics.

Data are presented as n (%) unless otherwise indicated.

^a^ Statistical significance was set at a 2-sided P<0.05.

^b^ Cardiovascular comorbidities include ischemic heart disease, hypertension, heart failure and atrial fibrillation.

The proportion of patients with severe/uncontrolled disease was significantly higher in COPD (20.0%) and ACO (25.3%) patients compared to asthma patients (9.7%), p value < 0.001. The majority of asthma patients were using ICS with LABA (89.5%), while ICS with LABA with ATC combination was the most prevalent regimen in COPD and ACO patients (p value <0.001). Patients with asthma had significantly less respiratory exacerbations in 2015 (7.3%) compared to COPD (14.4%) and ACO patients (17.2%), p value = 0.002.

### 3.2 Respiratory and all cause-related charges

Respiratory-related charges accounted for 48.7% of total all-cause health care charges for the asthma patients, 42.1% for the COPD and 44.6% for the ACO patients.

During the duration of the study, a total of 128 respiratory-induced hospital admissions were identified; 70 admissions were due to asthma exacerbations (0.14 per patient) whereas 38 (0.21 per patient) and 20 (0.23 per patient) admissions were due to COPD and ACO exacerbations, respectively. Additionally, the total number of the outpatient visits recognized during the study duration was 1,665 visits, classified as follows: 1,052 for asthma follow-up (2.1 per patient), 369 for COPD follow-up (2.1 per patient), and 244 for ACO follow-up (2.8 per patient). Total respiratory related charges, respiratory outpatient charges (per patient and per visit) were higher in patients with COPD and ACO compared to those with asthma, p values <0.001 (Table 2).

**Table 2:** Respiratory and all cause-related charges for the study population.

	Asthma	COPD	ACO	P value^a^
	Mean (SD)	Mean (SD)	Mean (SD)	
Average total respiratory related charges	354.3(604.7)	562.2(888.5)	601.35(815.9)	<0.001
Average respiratory related inpatient charges per patient	1046.1(1131.98)	1529.88(1512.14)	1338.86(1049.79)	0.142
Average charges per respiratory related inpatient visit	851.8 (852.4)	1046.76 (769.5)	937.2 (701)	0.119
Average charges per respiratory related inpatient day	238.2(205)	183.7(80)	192.2(100)	0.950
Average respiratory related outpatient charges per patient	214.94(205)	317.27(312)	360.3(329)	<0.001
Average charges per respiratory outpatient visit	77.6(61)	122.1 (94)	112.2(95)	<0.001
Average total all-cause charges	1251.72 (1881.2)	1705.4 (2136.47)	1830.76 (2466.56)	<0.001
Average inpatient charges per patient	1754.49 (2275.3)	1999.7 (2462.66)	1791.81(1899.1)	0.670
Average charges per inpatient visit	923.4 (1145.7)	1018.48 (1450.36)	931.743 (961.6)	0.440
Average charges per inpatient day	460.361 (732)	416.183 (651)	540.3 (955)	0.693
Average outpatient charges per patient	649.26 (894.459)	798.86 (636.917)	1027.53 (1692.48)	<0.001
Average charges per outpatient visit	57.9 (119.9)	68.06 (81)	68.9 (166)	<0.001

^a^ Charges were compared using Kruskal-Wallis test. The respiratory and all cause-related charges for the study population were calculated for the year 2016 and presented in Jordanian Dinars (JOD). 1 JOD = 1.410 USD.

The average all-cause charges in 2016 were found to be higher in patients with ACO and COPD compared to those with asthma (1830 and 1705, versus 1251 JODs, respectively, p value = 0.001) (Table 2). Average outpatient charges per patient were significantly higher in ACO patients compared with asthmatic patients (1027.53 versus 649.26 JODs, respectively, p value< 0.001). The average charges of the outpatient visit were slightly higher in the cases of COPD and ACO compared with asthma (68.06 and 68.9 versus 57.9 JODs, respectively, p value< 0.001) (Table 2).

For the total respiratory charges, geometric total charges were higher in ACO patients compared with asthma (ratio of 1.48, p = 0.010). The ratios of the geometric means were also statistically significant for older patient (age > = 65 years) and those with severe pulmonary function/uncontrolled condition (ratios of 1.35 and 1.68, respectively and adjusting for other potential confounders) (Table 3). Geometric total all-cause charges were higher in COPD and ACO patients compared with asthma (ratios of 1.37 and 1.49, respectively). Being in the group of older ages (> = 65 years), presenting with severe pulmonary function/uncontrolled condition and having CV comorbidities were also associated with higher total all-cause charges (ratios of 1.32, 1.41 and 1.62, respectively) (Table 3).

**Table 3:** Predictors of respiratory and all cause-related charges.

Variable	Coefficients	Exp (coefficients)	P value ^a^
**Total respiratory charge**			
Respiratory diagnosis			
Asthma	Ref		
COPD	0.1639	1.18	0.210
ACO	0.3887	1.48	0.010
Age ≥65	0.2968	1.35	0.004
Male gender	-0.0377	0.96	0.728
Severity indicator	0.5210	1.68	<0.001
CV comorbidities	0.0711	1.07	0.485
**Total all cause charge**			
Respiratory diagnosis			
Asthma	Ref		
COPD	0.3146	1.37	0.008
ACO	0.3986	1.49	0.005
Age (≥65)	0 .2759	1.32	0.003
Male gender	0.0293	1.03	0.766
Severity indicator	0.3451	1.41	0.005
CV comorbidities	0.4791	1.62	<0.001

Multivariable regression on log transformed charges. Variables were selected by backward stepwise process. Gender was added to all cause charges model for clinical importance.

For respiratory charges analysis, gender and CV comorbidities variables were included in the model for their clinical importance.

COPD: chronic obstructive pulmonary disease, ACO: Asthma-COPD overlap syndrome, CV: cardiovascular.

^a^ Statistical significance was set at a 2-sided P value < 0.05.

### 3.3 By-service charges analysis

By-service charges analysis for hospital admissions revealed that the charges of laboratory tests, drugs, residency, and procedures were the major charge driver components, which were relatively higher for the COPD and ACO patients compared to asthma patients (Table 4). In the outpatient scenario, the charge component attributed to medications was the underlying cause of the increase in the overall outpatient management of COPD and ACO conditions compared with asthma; 273.2 and 299 versus 169.7 JOD, respectively (Table 4).

**Table 4:** By service charges for respiratory induced admissions and outpatient visits per patient.

**By service charges for respiratory induced admissions per patient in 2016 [JOD].**			
	**Asthma**	**COPD**	**ACO**
	**n = 57**	**n = 26**	**n = 14**
Total (average (SD))	1046.1 (1131.9)	1529.9 (1512.1)	1338.9 (1049.8)
Lab	199 (243.9)	398.1 (441.9)	319.8 (262.2)
Drugs	237.3 (367.06)	334.4 (293.4)	290.1 (302.6)
Residency	175.0 (216.9)	313.8 (333.5)	297.6 (216.3)
Supervision	62.6 (82.7)	107.7 (86.6)	105.9 (77.9)
Procedures	73.3 (119.4)	171.7 (308.9)	186.6 (220.2)
Radiology	23.3 (39.0)	110.5 (190.4)	50.6 (89.2)
Consumables	93.7 (355.6)	84.9 (135.2)	75.6 (73.8)
Operation Fees	170.8 (416.2)	0.8 (3.2)	3.7 (8.2)
Consultation	7.6 (12.5)	7.3 (10.3)	6.8 (11.5)
Miscellaneous	3.0 (14.1)	0 .78 (3.7)	2.2 (8.1)
**By service charges of respiratory related outpatient visits per patient in 2016 [JOD].**			
	**Asthma**	**COPD**	**ACO**
	**n = 380**	**n = 142**	**n = 76**
Total (average (SD))	214.9 (204.9)	317.27 (312.40)	360.29 (329.612)
Drugs	169.7 (191.9)	273.2 (298.4)	299 (302)
Clinic Visit	22.0 (13)	20.4 (13)	25 (16)
Radiology	6.2 (18)	8.3 (24)	9.8 (22)
Lab	11.5 (30)	6.2 (21)	13.8 (35)
Procedures	5.5 (13)	9 (11)	12 (19)
Consumables	0.13 (1.3)	0.05 (0.4)	0.7 (4.6)

The service charges for respiratory induced admissions and outpatient visit were calculated for the year 2016 and is presented in JOD. 1 JOD = 1.410 USD.

^a^ Statistical significance was set at a 2-sided P value < 0.05.

**Fig 1:**
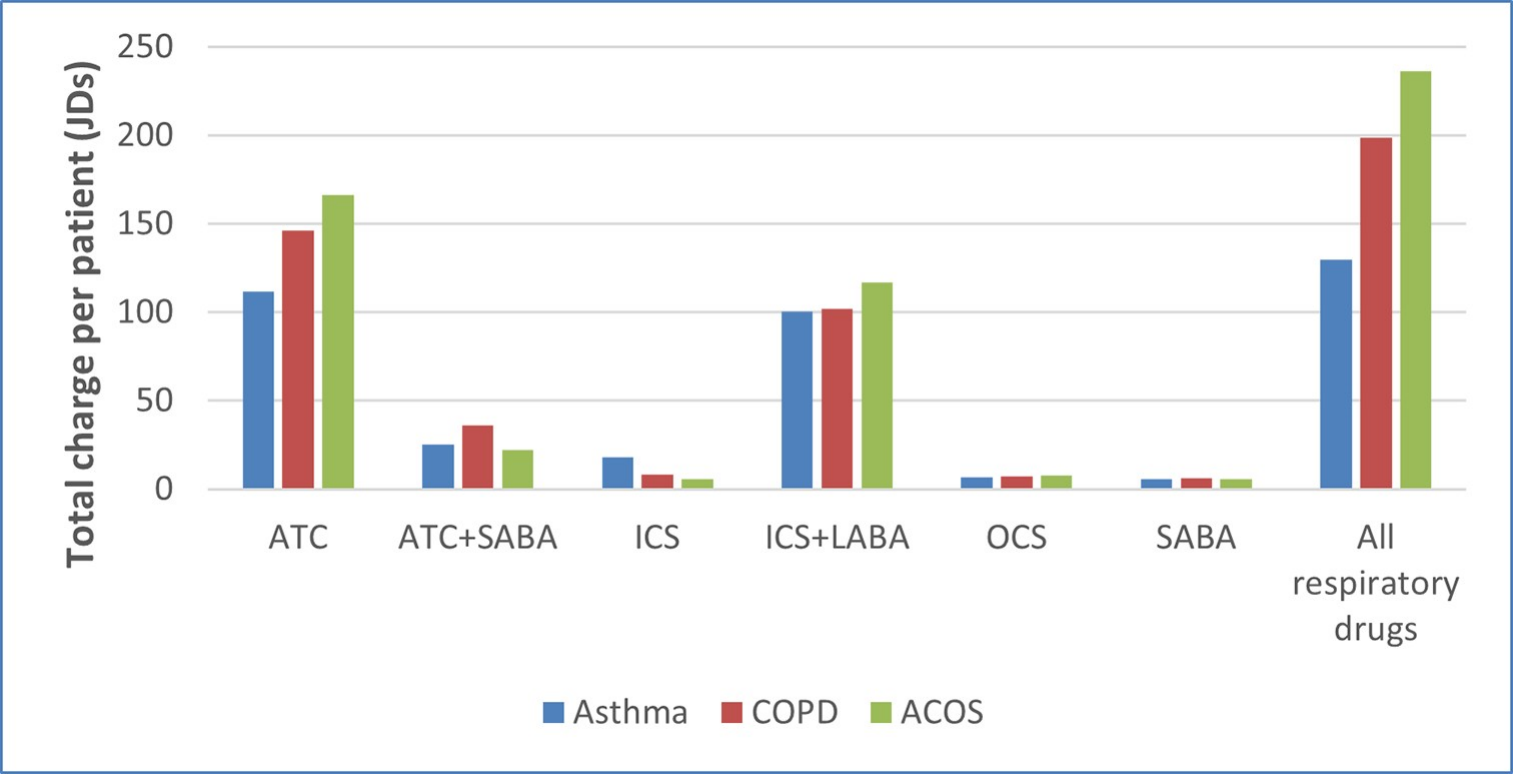
Per patient charges of respiratory drugs by disease.

Respiratory-related medication charges were a considerable portion of the total respiratory-related health care charges; approximately 65.5% for the asthma patients, 64.1% for the COPD patients and 64.2% for the ACO patients. Average per patient respiratory medication charges were the highest in ACO patients with ATC and ICS+LABA contributing to this increase in medication charges in ACO versus Asthma and COPD patients (Fig 1). Average all-cause medication expenditures per patient was also the highest in ACO patients compared to asthma and COPD patients (451.735 JOD versus 281.261 JOD and 378.023 JOD, respectively).

## 4 Discussion

In the current study, an economic analysis was conducted to estimate the burden of management of three respiratory disorders: asthma, COPD, and the overlap syndrome- ACO. The study data suggest that ACO is associated with considerable clinical and economic burden. Overall, patients with ACO had a higher disease severity compared to asthma and COPD patients. These results go in line with previous studies which have shown that the overlap syndrome is associated with more severity compared to either disease alone [10, 20, 21]. The rate of respiratory related exacerbations was also shown in previous studies to be higher in patients with ACO compared to patients with asthma or COPD [22–24].

Respiratory-related charges, representing almost 50% of total all-cause charges, were highest among the patients with ACO compared with asthma patients; a modest increase was shown when results were compared with COPD patients. Several previous studies revealed similar findings concerned with high respiratory expenditures, specifically in patients with ACO [13, 22, 25, 26]. One of the studies that was aimed to compare the respiratory-related costs between COPD and ACO found that costs of treatment of ACO patients were significantly higher than that for the COPD patients [26]. Shaya et al. studied the burden of concomitant asthma and COPD conditions in a Medicaid population and showed that patients with ACO consume more medical resources than either disease alone [25]. In the current study, having ACO versus asthma, being amongst the older age groups, and having higher disease severity were found to predict higher respiratory related charges. Similarly, ACO was found to independently influence healthcare utilization in previous literature [11, 13, 25, 27]. Gerhardsson de Verdier et al. showed that asthma-related costs were nearly twice as high among patients with ACO compared to patients with asthma [11]. In addition, the more the lung functions were found to be deteriorated, the more vigorous interventions were found to be needed, and the more expensive the treatment courses were.

Results from the current study showed that older ages are associated with higher respiratory expenditures. Previous studies estimated that 90% of patients misused the inhalers, and older patients were more liable to such misuse, due to many factors including physical and cognitive impairments, and the inability to have the needed skills [28, 29]. In the same context, studies have shown that improper use of inhalers and poor inhalation technique can eventually increase the pressure on both the healthcare providers and patients themselves [28, 30].

The all-cause charges in this study were measured by adding all expenses for the management of the diseases (asthma, COPD and ACO) including charges related to the respiratory condition itself, and other conditions and comorbidities. Results showed that the average all-cause charges were higher in the cases of ACO and COPD compared with the average charges incurred by asthmatic patients. Having ACO or COPD versus asthma, being in the older age groups, having CV comorbidities, and higher disease severity were found to predict higher all-cause charges in the current study. In a retrospective cohort analysis that used administrative claims data obtained from the HealthCore Integrated Research Environment (HIRE), results showed that all-cause health care costs were twice as high for patients with ACO compared to patients with asthma [11]. Kim et al. showed that ACO patients incurred the highest medical costs compared to asthma and COPD patients, while inpatient medical costs were the highest in COPD patients [27]. In the current study, no statistically significant difference was found regarding the in total all-cause charges between ACO and COPD patients. In fact, compared to asthma, COPD and ACO patients showed higher prevalence of comorbidities, including cardiovascular and diabetes mellitus comorbidities, which may have contributed to the greater utilization of the medical services and increased expenditures seen in these patients. In addition, the average age of patients with COPD and ACO was found to be relatively higher than that for patients with asthma (65 and 64, compared with 58, respectively). The demographic features of these diseases were similar as reported by other studies [22, 27]. Being older in age might indicate incorrect use of medications, specifically inhaler devices, and non-adherence, eventually leading to more exacerbations and uncontrolled conditions. In the outpatient setting, it was found that patients with ACO condition cost the healthcare system annually much more than COPD and asthma. The average charge per ACO patient attributed to all-cause outpatient services was almost 30% and 60% higher compared to COPD and Asthma, respectively.

Although this study showed minimal differences when ACO was compared with COPD in the previous aspects, it was obviously shown that ACO can still be considered of high disease burden and high health resource utilization. This is specifically true since the number of patients with ACO included in this study was relatively small compared with the total population number.

Results from the current study showed that respiratory-related medications contributed to more than 50% of total respiratory-related health care expenditures. Further, per patient respiratory medication charges were the highest in ACO patients compared to asthma and COPD patients. High medication expenditures in ACO patients were reported in previous studies; a study conducted in Taiwan showed that the average cost of most respiratory related medications per patient was the highest in ACO patients compared to asthma and COPD patients. ICS/LABA combinations were prescribed 3.15 times more frequently in patients with ACO than COPD, and ATC monotherapy was prescribed 9.5 times more frequently in patients with ACO than asthma [22]. Another study showed that the total cost of respiratory related medications was higher in ACO patients as compared to COPD (without asthma) patients. The total cost of each medication was also higher in ACO patients than the COPD alone patients [13].

When it comes to medications, ATC, and ICS+LABA were used more intensively in ACO patients versus asthma and COPD patients. This finding is of interest because of the dearth of data found regarding medication patterns for ACO compared to either disease alone. Yet, this outcome was reasonably expected considering that the ICS+LABA and ATC have always been the cornerstones in asthma and COPD management, respectively, and so both would be expected to be of value in the overlap syndrome, ACO. Of noteworthy, both treatments were used more intensively in ACO than that used for either disease alone, which is correlated with the higher disease severity in ACO patients compared to COPD and asthma patients.

In the current study, in addition to respiratory related medications, the average all-cause medication expenditures were shown to be highest in ACO patients. The higher presence of comorbidities in patients with ACO could be the main reason behind this finding.

### 4.1 Limitations

This study has some limitations. All patients’ information were obtained retrospectively from the medical records and pharmacy data of one hospital in northern Jordan. Missing some information regarding respiratory function tests imposed a challenge to determine the disease severity in the study sample. Pulmonary function test results were not available in medical records for a considerable percent of the study sample. However, respiratory exacerbations in the previous year were extracted and considered in determining the disease severity. The approach used to determine the disease severity in the current study was adopted in a previous study [19]. In addition, smoking status and BMI were missing for some patients. Other variables such as educational level and socioeconomic status were also not available in medical records, which could have added a further insight to the results. Only direct medical charges in the hospital setting were included in the current study. Other losses such as indirect and intangible costs were not assessed, still hospital expenses represent a major component. Patients with selected comorbidities such as psychiatric disorders and other respiratory diseases such as lung cancer and bronchiectasis were excluded, which may limit the generalizability of results to these patients. Direct charges of hospital admissions are difficult to allocate to one disease alone, however, the ICD10 codes reflect the major diagnosis in the admission and accordingly the major driver for expenditures. Only non-fatal cases were included in this study. Fatal cases may be more or less expensive. Further research is needed to evaluate the burden associated with mortality in this population. Using charges as a proxy to estimate costs is practical and informative, however it is not free from criticism [31]. Charges of public healthcare services in Jordan make up around 20% or less of the actual cost [32]. Finally, misdiagnosing ACO and the failure to diagnose COPD in patients with asthma is definitely a concern, especially in older adults [33]. Future prospective research is needed to effectively capture and characterize patients with ACO.

## 5 Conclusions

ACO is associated with considerable clinical and economic burden in Jordan. Patients with ACO had a higher disease severity compared to asthma and COPD patients. Older ages, higher disease severity, and cardiovascular comorbidities are associated with higher health care expenditures.
